# ADVANCING THE SCIENCE OF SENSORY HEALTH AND COGNITION: FROM EPIDEMIOLOGY TO INTERVENTION

**DOI:** 10.1093/geroni/igae098.1995

**Published:** 2024-12-31

**Authors:** Carrie Nieman, Jennifer Deal

**Affiliations:** Chair; Discussant

## Abstract

Sensory health and cognition are at the intersection of two major public health challenges facing an aging global population. Sensory impairments are among the most common and disabling comorbidities among individuals at risk for cognitive impairment and those already aging with cognitive impairment. Sensory impairments may also serve as key biomarkers in dementia and may worsen the trajectory of decline. Although prevalent, sensory impairments frequently go unrecognized and unaddressed. Importantly, sensory impairment has been identified as modifiable risk factors for dementia. Optimizing sensory function may be an important yet overlooked approach to reducing the risk of cognitive decline as well as providing potential nonpharmacological interventions to aid in the management of neuropsychiatric symptoms, improve quality of life for persons living with dementia, and reduce burden for care partners. This symposium presents the latest evidence from epidemiology to intervention in advancing our understanding and approach to sensory health and cognition. The symposium begins with a focus on the epidemiology of sensory function among individuals with or at risk for cognitive impairment and then moves to a focus on intervention, understanding the potential impacts of optimizing sensory function on brain structure, barriers to addressing sensory function in clinical settings, and envisioning how to optimize interventions by learning directly from individuals aging with cognitive impairment, their care partners, and experts. This symposium features leaders in the field of sensory health and cognition from diverse career stages, disciplines, and settings.

